# Residential exposure to radon and DNA methylation across the lifecourse: an exploratory study in the ALSPAC birth cohort

**DOI:** 10.12688/wellcomeopenres.14991.2

**Published:** 2019-04-15

**Authors:** Frank de Vocht, Matthew Suderman, Alberto Ruano-Ravina, Richard Thomas, Richard Wakeford, Caroline Relton, Kate Tilling, Andy Boyd

**Affiliations:** 1 Population Health Sciences, Bristol Medical School, University of Bristol, Bristol, UK; 2 MRC Integrative Epidemiology Unit, University of Bristol, Bristol, UK; 3 University of Santiago de Compostela, Santiago de Compostela, Spain; 4 Avon Longitudinal Study of Parents and Children, University of Bristol, Bristol, UK; 5 Centre for Occupational and Environmental Health, University of Manchester, Manchester, UK

**Keywords:** ALSPAC, Radon, DNA methylation, epigenetics. ARIES, geo-spatial epidemiology

## Abstract

**Background:** Radon (and its decay products) is a known human carcinogen and the leading cause of lung cancer in never-smokers and the second in ever-smokers. The carcinogenic mechanism from radiation is a combination of genetic and epigenetic processes, but compared to the genetic mechanisms, epigenetic processes remain understudied in humans. This study aimed to explore associations between residential radon exposure and DNA methylation in the general population.

**Methods:** Potential residential radon exposure for 75-metre area buffers was linked to genome-wide DNA methylation measured in peripheral blood from children and mothers of the Accessible Resource for Integrated Epigenomic Studies subsample of the ALSPAC birth cohort. Associations with DNA methylation were tested at over 450,000 CpG sites at ages 0, 7 and 17 years (children) and antenatally and during middle-age (mothers). Analyses were adjusted for potential residential and lifestyle confounding factors and were determined for participants with complete data (n = 786 to 980).

**Results:** Average potential exposure to radon was associated in an exposure-dependent manner with methylation at cg25422346 in mothers during pregnancy, with no associations at middle age. For children, radon potential exposure was associated in an exposure-dependent manner with methylation of cg16451995 at birth, cg01864468 at age 7, and cg04912984, cg16105117, cg23988964, cg04945076, cg08601898, cg16260355 and cg26056703 in adolescence.

**Conclusions: **Residential radon exposure was associated with DNA methylation in an exposure-dependent manner. Although chance and residual confounding cannot be excluded, the identified associations may show biological mechanisms involved in early biological effects from radon exposure.

## Introduction

Radon is a noble gas with no stable isotopes. Radon-222 (half-life (t _1/2_) 3.82 days) and radon-220 (t _1/2_ 55.2 seconds) are found in the environment as components of the radioactive decay chains of the naturally occurring, long-lived radionuclides uranium-238 and thorium-232, respectively, which are found to varying extents in all rocks and soil. Radon-222 is the product of the decay of long-lived radium-226 (t _1/2_, 1600 years), and in most parts of the world, following their accumulation in enclosed spaces, inhalation of ^222^Rn and its short-lived radioactive decay products is the largest contribution of human exposure to ionising radiation. Globally, inhalation of ^222^Rn and its progeny is estimated to provide nearly half of the average annual effective dose (the radiation- and tissue-weighted whole-body absorbed dose) of 2.4 mSv from natural sources of ionising radiation ^1^. However, the geographical variation of the effective dose from ^222^Rn and its progeny is considerable, with a typical range of 0.2-10 mSv per annum. The contribution to the global average annual effective dose from the inhalation of ^220^Rn and its progeny is much less at 0.1 mSv.

Radon-222 and some of its short-lived progeny deliver most of their radiation dose through short-range alpha-particle emission and, following inhalation, the radiation dose is received primarily by the bronchial epithelium from radon decay products. There is compelling epidemiological and experimental evidence that ^222^Rn and its decay products (hereafter, “radon”) cause lung cancer, with exposure-response associations approximately linear with no evidence of a threshold ^2, 3^, and radon has been classified as a Group 1 carcinogen (“carcinogenic to humans”) by the International Agency for Research on Cancer (IARC) ^4^.

Exposure to radon is considered the second leading cause of lung cancer after tobacco smoking, and the principal cause in never-smokers ^5, 6^. The fraction of the lung cancer burden attributable to indoor exposure to radon ranges from 3% to 14% across the world, and is estimated at 3.3% ^7^, or 1,100 lung cancer deaths annually, in the UK specifically ^3^. Once inhaled, radon gas itself is mostly exhaled again, but a large proportion of the inhaled short-lived radon progeny deposits in the airways of the lungs with the alpha-particles emitted by ^218^Po and ^214^Po dominating the dose to the lung. In contrast, radon gas transported from the lung makes a larger contribution than its decay products to doses to organs/tissues other than the lung, particularly those with a comparatively high fat content (including the red bone marrow (RBM)). However, the evidence for radon causing cancers other than lung cancer is limited and relates to the fact that doses to other tissues from radon are relatively small. For example, the UK average annual equivalent dose (the radiation-weighted absorbed dose) to the RBM from radon is 80 µSv (children and adults) as compared to the RBM dose of 1430 µSv (5-year old) and 1070 µSv (adult) from all-natural sources ^8^; this RBM equivalent dose from radon compares with that to the lung of 10,000 µSv.

Worldwide, the population-weighted geometric mean indoor level of radon activity concentration is estimated to be 30 Bq m ^-3^ ^9^, with a large geographical variation ^3^. In England, the concentration in homes is about 20 Bq m ^-3^ on average, but it ranges from 5 to 10,000 Bq m ^-3^ and more in some radon-prone areas; for comparison, the average outdoor concentration is 4 Bq m ^-3^ ^2^. Variation between and within small geographical areas, as well as over time, can be the result of many factors including the abundance of ^226^Ra in the ground, fissuring of rocks, permeability of the soil, openings in the foundations of buildings through which radon can enter, and the extent to which a particular structure retains radon, including ventilation ^3, 10^. In Great Britain, a strong correlation between domestic radon levels and socio-economic status (SES) has been observed, where lower SES residences have, on average, only two-thirds of the radon levels of those of the more affluent, which may be related to greater underpressure in warmer and better-sealed houses ^11^. Because people spend a significant portion of their time indoors, homes are typically the primary source of indoor radon exposure ^3^, and within houses concentrations can also widely vary, with (in the USA) concentrations typically 50% higher in basements compared to the ground floor ^12^.

The World Health Organisation (WHO) and International Commission on Radiological Protection (ICRP) recommend radon reference levels for homes in the range of 100-300 Bq m ^-3^ ^13^, with the ICRP reference level of 300 Bq m ^-3 ^having been incorporated as the upper limit for the reference level by the European Union ^14^. The annual effective dose for a dwelling at 300 Bq m ^-3^, and given several assumptions, is estimated at about 14 millisievert (mSv) ^15^. In the UK, Public Health England recommends that indoor radon levels should be below 200 Bq m ^-3^ (averaged over the home; the Action Level), which corresponds to about 12 mSv annual effective dose ^2^, with 100 Bq m ^-3^ being considered the Target Level for remediation work and for new buildings ^2^.

The multistage carcinogenic process is in all probability a mixture of genetic and epigenetic processes. Ionizing radiation, in addition to producing mutations mainly by gene deletion and gross chromosomal damage, can also induce epigenetic effects ^4^. Residential radon exposure has been associated with DNA-repair gene polymorphisms in adults ( *XpG* gene Asp1104His, *ADPRT* gene Val762Ala, and *NBS1* gene Glu185Gln polymorphisms) ^16^ and partly replicated in children ( *XpD* gene Lys751Gln, *XpG* gene Asp1104His and *ADPRT* gene Val762Ala polymorphisms) ^17^, with the latter study also reporting double-strand break repair gene polymorphisms. Epigenetics describe heritable chemical modifications of DNA and chromatin affecting gene expression, and include DNA methylation, histone modifications and microRNAs which can act in concert to regulate gene expression ^18^. In addition, the ‘bystander effect’, in which cells that are not directly irradiated, but are in the neighbourhood of cells that have, also exhibit phenotypic features of genomic instability that is considered to be epigenetic in nature ^4^. DNA methylation is the most stable and most readily quantifiable epigenetic marker and is sensitive to pre- and post-natal exogenous influences ^19^. Although the mechanisms of radiation-induced changes in DNA methylation remain largely unknown, the most plausible mechanism that has been proposed describes the effects of radiation on DNA methyltransferases ^20^, while it has further been suggested that low dose radiation can increase DNA methylation at least in part through the generation of Reactive Oxygen Species (ROS) ^21, 22^. Ionising radiation exposure has been shown to affect DNA methylation in *in vivo* studies, and which has the potential to be transmitted via the germline to subsequent generations ^23, 24^. However, there is only limited data on effects of radon exposure on DNA methylation in humans, with some evidence from high exposed uranium miners in China ^25^.

This study aims to explore whether there is evidence of DNA methylation from residential radon exposure in the general population and assesses whether any methylation varies across the lifecourse.

## Methods

### Data

This study used data from the Avon Longitudinal Study of Parents and Children (ALSPAC) ^26, 27^. ALSPAC recruited 14,541 pregnant women with expected delivery dates between April 1991 and December 1992, which resulted in 14,062 live births of which 13,988 children were alive at 1 year of age. Details of all data searchable though are provided at the ALSPAC data dictionary.

A sub-sample of 1,018 ALSPAC mother–child pairs had DNA methylation measured using the Infinium HumanMethylation450 BeadChip (Illumina, Inc.) ^28^ as part of the Accessible Resource for Integrated Epigenomic Studies (ARIES) project ^29^. For this study DNA methylation data generated from cord blood, venous blood samples at age 7 years and again at age 15 or 17, and additionally from the mothers during pregnancy and at middle age were used. All DNA methylation analyses were performed at the University of Bristol as part of the ARIES project and has been described in detail previously ^29^.

Ethical approval for this study was obtained from the ALSPAC Ethics and Law Committee and the Local Research Ethics Committees (Reference B2805).

### DNA methylation

DNA methylation profiles for ALSPAC children were generated using the Illumina Infinium HumanMethylation450 BeadChip as part of the Accessible Resource for Integrated Epigenomic Studies (ARIES) ^29^. DNA was bisulphite-converted using the Zymo EZ DNA Methylation ^TM^ kit (Zymo, Irvine, CA). Infinium HumanMethylation450 BeadChips (Illumina, Inc.) and used to measure genome-wide DNA methylation levels at over 485,000 CpG sites. The arrays were scanned using an Illumina iScan, with initial quality review using GenomeStudio (version 2011.1). This assay detects methylation of cytosine at CpG islands using one probe to detect the methylated and one to detect the unmethylated loci. Single-base extension of the probes incorporated a labelled chain-terminating ddNTP, which was then stained with a fluorescence reagent. The ratio of fluorescent signals from the methylated site versus the unmethylated site determines the level of methylation at the locus.

Quality control and normalization of the profiles was performed using the meffil R package (version 1.1.0) as previously described ^30^. The level of methylation is expressed as a percentage (β-value) ranging from 0 (no cytosine methylation) to 1 (complete cytosine methylation). Finally, to reduce influence of outliers in statistical models, normalized β-values were 90%-Winsorized.

### Radon exposure

Potential residential radon exposure is available from the Health Protection Agency (HPA; now Public Health England) – British Geological Survey (BGS) ‘radon potential dataset for Great Britain’, and was obtained for the Avon area (which includes the original ALSPAC catchment area) from BGS after a data sharing agreement was agreed by BGS and the PI’s Institute. Estimates of potential radon exposure were based on long-term radon measurements from 479,000 homes across Great Britain and provided with a spatial resolution of 75-metre buffers as the percentage of dwellings exceeding the 200 Bq m ^-3^ Radon Action Level in 6 classes: 1 (0-1%), 2 (1-3%), 3 (>3-5%), 4 (>5-10%), 5 (>10-30%) and 6 (>30-100%). More information is available at: http://www.bgs.ac.uk/radon/hpa-bgs.html. To assess measurement error, we also linked the ARIES dataset to estimates from the freely available radon ‘indicative atlas’ ^31^, which is based on the ‘potential dataset’, but provides the estimates in 1-km-size squares.

Residential histories of mothers and children were geocoded to postcode centroid level, and were linked to average potential radon exposure using ArcGIS software (version 10.6) ^32^ within the ALSPAC Data Safe Haven. This resulted in at least one address match for 986 mothers and 1001 young people (including two sets of twins). Once each residential address had a radon potential exposure class assigned, time spent at each address was calculated. This was merged with ARIES sample prevision dates, allowing time-weighted average potential radon exposures to be calculated up to the ‘mothers at middle age’, ‘children at 7’ and ‘children at 15/17’ sample extraction time points. For the cord and antenatal sample extractions, radon exposure potential of address at date of birth or closest address (temporally) to sample time point were assigned respectively.

These data were then linked to ALSPAC self-reported data selected to test for potential confounding (described below). After the linked exposure data were processed to minimise the risk of participant disclosure, the linked methylation-radon data were used for statistical analyses.

### Statistical methods

For these analyses we only use participants with complete data. In the primary analyses average potential radon exposure was analysed as a continuous variable (range 1–6) to assess linear exposure-response associations. In addition, we also analysed associations based on binary exposure classifications (≤5% vs >5%).

Associations were tested using linear models using the limma R package (version 3.32.10) ^33^. Associations were tested in (1) univariate analyses but with adjustment for the surrogate variables ^34^ to handle batch effects, sex differences, cell count heterogeneity and possible unknown confounders ^35^, and (2) additionally with adjustment for potential confounding factors maternal age at birth, maternal BMI, smoking during pregnancy, partner smoking during pregnancy, *AHRR* CpG site that detects own smoking nearly as accurately as self-report ^36^, mother alcohol intake in early pregnancy, equivalized income, parental occupation, and parental education, and (3) all factors of models 1 and 2 and additionally for damp problems, central heating, boiler location, gas cooking, time windows open in the summer/winter day/night, and heavy traffic.

Because of the exploratory nature of this study, associations at false discovery rate (FDR) less than 20% calculated using the *q* method ^37^ are reported instead of a more traditional 10% or 5% threshold. Where associations were positive, these sites were defined as “hypermethylated” and conversely when for inverse associations, these were defined as “hypomethylated”.

## Results

### Participants and location

**Figure 1.**
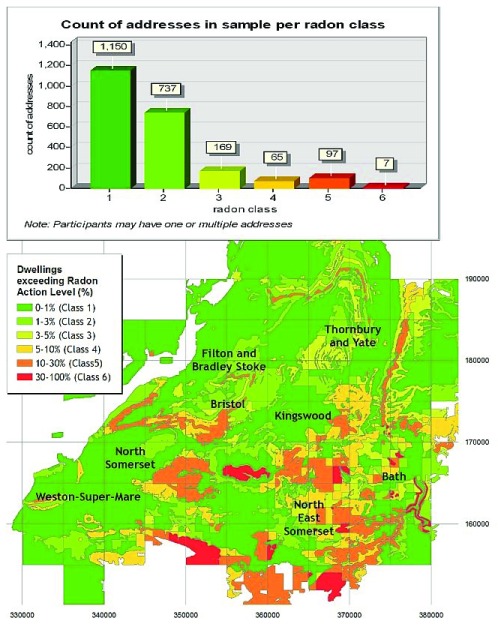
Geographical distribution of potential radon exposure classes, and number of addresses per class. Based upon the ‘Radon Potential Dataset’, reproduced with the permission of the British Geological Survey.

The results were based on 786 to 980 participants with complete information, depending on the time analysed. A graphical overview of the geographical study area and the distribution of potential radon exposure classes, as well as the distribution of addresses in each class, is shown graphically in Figure 1, and indicates that 79% of addresses are in areas with low probability (class 1 and 2) of exposure >200 Bq m ^-3^.

### CpG sites

Results for CpG sites with FDR <0.20 are shown in Table 1. In mothers, average potential exposure to radon was only associated in an exposure-dependent manner with hypomethylation of cg25422346 during pregnancy (p = 1.1x10 ^-8^, FDR = 0.005), with no associations observed at middle age. For the children, radon potential exposure was associated in an exposure-dependent manner with hypomethylation at cg16451995 at birth (p = 3.2x10 ^-7^, FDR = 0.16) and with hypermethylation of cg01864468 at age 7 (p = 1.1x10 ^-8^, FDR = 0.005). In adolescence (age 15–17) there was evidence of exposure-dependent methylation at several CpG sites. Cg04912984, cg16105117 and cg23988964 were hypermethylated with increased potential exposure, while cg04945076, cg08601898, cg16260355 and cg26056703 were hypomethylated proportionally to average potential radon exposure. The same CpG sites at the same timepoints were identified when average potential exposure to >200 Bq m ^-3^ was dichotomized into low (≤5%) and high (>5%) probability ( Table 2), and similarly when using another cut-off (≤3% vs >3%); data not shown.

**Table 1.** Potential exposure to >200 Bq m ^-3^ radon and methylation at specific CpG sites.

CpG	Chromosome	Gene	N (Model 1)	Model 1 ^1^ Beta (se)	N (model 2)	Model 2 ^2^ Beta (se)	N (model 3)	Model 3 ^3^ Beta (se)	Min. FDR (adjusted)
**Mothers during pregnancy**									
cg25422346	4	upstream SMIM31	980	-0.002 (0.001)	712	-0.002 (0.001)	630	-0.003 (0.001)	0.005
**Cord blood**									
cg16451995	16	PMM2	912	-0.005 (0.001)	649	-0.005 (0.001)	576	-0.004 (0.001)	0.123
**Age 7**									
cg01864468	6	upstream HCG14	978	0.006 (0.002)	697	0.004 (0.002)	615	0.002 (0.002)	0.173
**Age 15 ^4^**									
cg04912984	1	upstream VANGL1	979	0.002 (0.001)	313	0.003 (0.001)	313	0.004 (0.001)	0.184
cg04945076	12		979	-0.007 (0.002)	313	-0.008 (0.003)	313	-0.008 (0.004)	0.184
cg08601898	8		979	-0.002 (0.001)	313	-0.004 (0.002)	313	-0.004 (0.002)	0.184
cg16105117	14	NDRG2	979	0.000 (0.000)	313	0.000 (0.000)	313	0.000 (0.000)	0.185
cg16260355	10	SGPL1	979	-0.001 (0.001)	313	0.000 (0.001)	239	-0.000 (0.001)	0.093
cg23988964	1	upstream FAM71A	979	0.004 (0.001)	313	0.001 (0.002)	239	0.002 (0.003)	0.185
cg26056703	15	LINC01197	979	-0.001 (0.001)	313	-0.000 (0.001)	239	-0.000 (0.001)	0.184
**Mothers at middle age**									
None									

^1^Adjusted for surrogate variable only. ^2^Adjusted for surrogate variable, maternal age at birth, maternal BMI, smoking during pregnancy, partner smoking during pregnancy, mother alcohol intake in early pregnancy, equivalized income, parental occupation, parental education. ^3^Adjusted for surrogate variable, maternal age at birth, maternal BMI, smoking during pregnancy, partner smoking during pregnancy, mother alcohol intake in early pregnancy, equivalized income, parental occupation, parental education, damp problems, central heating, boiler location, gas cooking, time windows open in the summer/winter day/night, heavy traffic. ^4^At age 15/17, also adjusted for AHRR CpG site that detects own smoking.

**Table 2.** CpG site methylation and low (≤5%) vs high (>5%) probability of potential exposure to >200 Bq m ^-3^ radon (FDR<0.20).

CpG	Chromosome	Gene	N (Model 1)	Model 1 ^1^ Beta (se)	N (model 2)	Model 2 ^2^ Beta (se)	N (model 3)	Model 3 ^3^ Beta (se)	Min. FDR (adjusted)
**Mothers during pregnancy**									
cg25422346	4	upstream SMIM31	980	-0.012 (0.002)	712	-0.010 (0.002)	630	-0.011 (0.003)	0.005
**Cord blood**									
cg16451995	16	PMM2	912	-0.020 (0.004)	649	-0.018 (0.004)	576	-0.016 (0.005)	0.123
**Age 7**									
cg01864468	6	upstream HCG14	970	0.017 (0.006)	692	0.016 (0.008)	611	0.012 (0.009)	0.173
**Age 15 ^4^**									
cg04912984	1	upstream VANGL1	965	0.013 (0.003)	310	0.014 (0.005)	236	0.020 (0.005)	0.184
cg04945076	12		965	-0.037 (0.007)	310	-0.032 (0.012)	236	-0.029 (0.014)	0.184
cg08601898	8		965	-0.018 (0.004)	310	-0.031 (0.006)	236	-0.031 (0.007)	0.184
cg16105117	14	NDRG2	965	0.003 (0.001)	310	0.002 (0.001)	236	0.002 (0.001)	0.185
cg16260355	10	SGPL1	965	-0.007 (0.002)	310	-0.008 (0.004)	236	-0.009 (0.005)	0.093
cg23988964	1	upstream FAM71A	965	0.026 (0.006)	310	0.010 (0.008)	236	0.010 (0.009)	0.185
cg26056703	15	LINC01197	965	-0.014 (0.003)	310	-0.009 (0.005)	236	-0.014 (0.005)	0.184
**Mothers at middle age**									
None									

*Methylation for population with average potential radon exposure 5% or lower compared with population with probability >5%. ^1^adjusted for surrogate variable only. ^2^adjusted for surrogate variable, maternal age at birth, maternal BMI, smoking during pregnancy, partner smoking during pregnancy, mother alcohol intake in early pregnancy, equivalized income, parental occupation, parental education. ^3^Adjusted for surrogate variable, maternal age at birth, maternal BMI, smoking during pregnancy, partner smoking during pregnancy, mother alcohol intake in early pregnancy, equivalized income, parental occupation, parental education, damp problems, central heating, boiler location, gas cooking, time windows open in the summer/winter day/night, heavy traffic. ^4^At age 15/17 also adjusted for AHRR CpG site that detects own smoking.

Regardless of exposure metric, there is little evidence of significant confounding with directions and sizes of associations, similar for univariable and both multivariable models.

To assess the impact of measurement error, the same analyses were repeated but with exposure based on the ‘indicative radon atlas’ using 1-km ^2^ spatial resolution ( Table 3). Results were comparable to those based on the 75-m buffers.

**Table 3.** Indicative (1 km ^2^ spatial granularity) potential exposure to >200 Bq m ^-3^ radon and CpG site methylation.

CpG	Chromosome	Gene	N (Model 1)	Model 1 ^1^ Beta (se)	N (model 2)	Model 2 ^2^ Beta (se)	N (model 3)	Model 3 ^3^ Beta (se)	Min. FDR (adjusted)
**Mothers during pregnancy**									
cg25422346	4	upstream SMIM31	857	-0.001 (0.001)	615	-0.001 (0.001)	543	-0.002 (0.001)	0.005
**Cord blood**									
cg16451995	16	PMM2	786	-0.004 (0.001)	551	-0.004 (0.001)	485	-0.003 (0.001)	0.123
**Age 7**									
cg01864468	6	upstream HCG14	851	0.009 (0.002)	596	0.007 (0.002)	524	0.006 (0.002)	0.173
**Age 15 ^4^**									
cg04912984	1	upstream VANGL1	852	0.001 (0.001)	272	0.002 (0.001)	208	0.004 (0.002)	0.184
cg04945076	12			-0.002 (0.002)	272	-0.010 (0.004)	208	-0.008 (0.005)	0.184
cg08601898	8		852	-0.002 (0.001)	272	-0.002 (0.002)	208	-0.004 (0.002)	0.184
cg16105117	14	NDRG2	852	0.000 (0.000)	272	0.000 (0.000)	208	0.000 (0.000)	0.185
cg16260355	10	SGPL1	852	-0.002 (0.001)	272	-0.000 (0.001)	208	-0.001 (0.002)	0.093
cg23988964	1	upstream FAM71A	852	0.001 (0.002)	272	0.002 (0.003)	208	0.001 (0.003)	0.186
cg26056703	15	LINC01197	852	-0.001 (0.001)	272	-0.000 (0.002)	208	-0.002 (0.002)	0.184
**Mothers at middle age**									
None									

^1^Adjusted for surrogate variable only. ^2^Adjusted for surrogate variable, maternal age at birth, maternal BMI, smoking during pregnancy, partner smoking during pregnancy, mother alcohol intake in early pregnancy, equivalized income, parental occupation, parental education. ^3^Adjusted for surrogate variable, maternal age at birth, maternal BMI, smoking during pregnancy, partner smoking during pregnancy, mother alcohol intake in early pregnancy, equivalized income, parental occupation, parental education, damp problems, central heating, boiler location, gas cooking, time windows open in the summer/winter day/night, heavy traffic. ^4^At age 15/17 also adjusted for AHRR CpG site that detects own smoking.

We used complete-case analyses which resulted in different numbers of subjects included in the different models. However, assessment of the impact of case deletion indicated little differences between the different populations used for models 1-3, with the possible exception of smoking of the father for outcomes at birth an age 7 ( *Extended data*: Supplementary tables ^38^).

## Discussion and conclusions

In this exploratory study we aimed to investigate associations between residential exposure to radon in the general population and DNA methylation. Associations were observed with increasing probability of average potential exposure of the residence over 200 Bq m ^-3^ in children at birth, age 7 and during adolescence, with single CpG sites affected at birth (cg16451995) and age 7 (cg01864468) and seven sites affected at age 15–17 (cg04912984, cg04945076, cg08601898, cg16105117, cg16260355, cg23988964, cg26056703) after adjustment for important confounding factors. These also did not depend on the choice of cut-off used. However, none of these associations were observed at multiple time points. For mothers an association with hypomethylation of cg25422386 was observed during pregnancy, but not at a later time point during middle age. To our knowledge, this is the first study identifying associations between radon exposure with methylation patterns in a general population.

Locations of the affected CpG sites of the children were on the *PMM2* gene (cord blood), associated with abnormalities in amniotic fluid and congenital disorders, upstream of *HCG14* (age 7), involved in the development of lung carcinoma, and *NDRG2* and *SGPL1*, associated with glioblastoma development and Alzheimer’s disease and nephrotic syndrome, respectively, *LINC01197,* as well as upstream of *VANGL1*, associated with congenital disorders, and *FAM71A* genes (age 15–17). For mothers cg25422346 is located upstream of *SMIM31*. These methylation patterns, describing both hyper- and hypo-methylation associated with potential residential radon exposure, have not been reported elsewhere. Data from *in vitro* experiments has similarly shown both hypo-and hyper-methylation, suggesting methylation status is probably dependent on the direct activity of the methylation machinery and could be mediated by the activity of associated DNA methyltransferases ^22^. Dose-dependent effects have also been demonstrated for global DNA methylation and *LINE-1* in nuclear power plant workers ^39^. In a candidate gene study of Chinese uranium miners, the authors reported increased methylation of promotor regions of *p16 ^INK4a^* and *O ^6^-MGMT* genes, as well as increased total methylation rate, depending on cumulative radon doses ^20^, and a study using BEAS-“B human lung cells exposed to 20,000 Bq m ^-3^ radon for 30 minutes showed global hypomethylation and hypermethylation of candidate CpG-sites at *PTPRM* and *EDA2R* genes ^40^. Similarly, these genes have also not appeared in candidate gene studies of exposure to radon, in which gene-environment interactions with *p53* ^41^, *GSTM1* and *GSTT1* ^42^, *hOGG1* and *APE1* ^43^, *ADPRT* ^44^, *XPG*, *ADPRT* and *NBS1* ^16^, *LIG4* ^45^, and *NBS1* and *ATM1* have been reported. Possible explanations for the different genes for which hyper- or hypomethylation was associated with potential radon exposure in this study compared to other studies may be that CpG sites identified in this study are involved in the ‘bystander effect’ rather than the result of direct irradiation, they may be a marker of earlier biological effects, it may be because methylation was measured in blood rather than in lung tissue, and of course residual confounding or chance findings also cannot be excluded.

This study has several limitations. Most importantly, the exposure metric used in this study is a relatively weak one. It is not generally possible to accurately predict indoor radon concentrations for specific buildings without individual measurements ^3^. Although people spend most of their time indoors at home, estimates are based on the modelled probability that a dwelling in the 75-m buffer that includes a person’s home has a radon concentration exceeding 200 Bq m ^-3^. Because of high spatial and temporal variability ^46, 47^ this will inevitably have led to considerable misclassification. Assuming measurement error in this case is non-differential, generally resulting in bias to the null, it is interesting that exposure-response associations were still observed in this study with a relatively small sample size. Furthermore, the possibility of misclassification of radon exposure should affect all participants in a similar way and is unlikely to bias associations with DNA methylation.

Although there was little evidence of significant confounding in these analyses, residual confounding as an explanation for these findings cannot be excluded. For example, rurality is a known confounding factor for studies on radon ^47^. However, within ALSPAC and certainly within Avon there are few true ‘rural’ residential areas as the area is quite heavily populated, so it is unlikely this will bias associations significantly. We also had no information on whether participants lived in houses or apartments (and in the latter case on which floor) ^46^ or whether houses had a basement ^12^, which will have added to further measurement error.

There are known limitations in quality of the ALSPAC residential address history data in terms of missingness and gaps; although in this study the impact of this will be limited as the postnatal ARIES sample dates are linked to direct contact with participants where address details would have been validated. However, to enable assignment of potential radon exposure to individuals over periods of unknown residence, remediation was carried out by (a) setting the address start-date to child date of birth where first address start-date fell after child date of birth, which is a reasonable assumption because often the address start-date represents a data capture date as opposed to an actual move date, and (b) by rectifying all other temporal gaps by calculating a mean radon potential exposure class based on the radon potential at the preceding and succeeding addresses.

The current analyses lack directly measured blood-cell-type proportions, and we therefore included cell count heterogeneity using estimates obtained using surrogate variable analysis in the models ^34^. This approach has been found to perform just as well or better ^35^ than the more commonly used method of Houseman et al. ^48^. In this case, it probably performs better in DNA methylation profiles generated from childhood peripheral blood because DNA methylation references are available only for adult blood ^49^ and cord blood. Levels of methylation vary between tissue types and may relate differently to traits and exposures, which may limit inferences from this study. In the current study we have methylation from blood samples, but it may have been beneficial had we been able to test associations in a more relevant cell type such as the lung.

This study had reduced statistical power due to the relatively limited sample size of the ARIES sub-sample, which was further diminished as a result of missing values. Alcohol consumption for adolescents could not be included as a potential confounding variable because this was only available for less than 200 teens. Because current approaches for multiple imputation are not feasible for genomic datasets including hundreds of thousands of measured variables (our study included variables corresponding to DNA methylation levels at over 480,000 CpG sites) we did not apply multiple imputation to increase sample size. In future, when feasible approaches have been developed, we plan to revisit these analyses.

Finally, because of the exploratory nature of this study we relaxed the FDR threshold for reporting of findings to 20% to minimise the probability of missing associations. The drawback of this choice was that some of our findings may have being false positives. Had a 10% FDR, more traditional in confirmatory studies, been used, only methylation at cg25422346 in mothers during pregnancy and of cg16260355 in adolescents would have been highlighted.

The main strength of this study is the unique resource which allowed for the assessment of genome-wide methylation profiles at different time points linked to detailed phenotypic characterisation, which enabled assessment of the temporality of associations. In these analyses we used three cross-sectional models to compare methylation patterns at birth, age 7 and in adolescence, but with better characterization of the dynamic elements of the human methylome ^50^, longitudinal analyses will help to better elucidate persistent and reversible effects of (environmental) exposures as well as critical periods of effect ^51^. Information on epigenetic signals across the life-course and radon exposure are of interest because they have the potential to describe early biological effects, and the estimated induction (lag) period of lung cancer to radon exposure is between 5 and around 25 years ^1^.

In conclusion, this exploratory study is, to our knowledge, the first study to examine the association between genome-wide DNA methylation and (potential) residential exposure to radon. Despite the relatively weak exposure metric, differential methylation associated with increased potential residential radon exposure was observed prenatally in mothers, for children at birth, age 7, and especially at age 15–17, but not for the mothers in middle age. Future work in a larger population, with replication in an independent sample, and using a more accurate radon exposure estimation methodology, most notably personal exposure measures, can further elucidate these associations.

## Data availability

### Underlying data

The potential residential radon exposure was provided by the British Geological Survey (BGS) under license for the current study (Licence number 2017/017RAD ED British Geological Survey © NERC. All rights reserved) and can be requested from BGS ( http://www.bgs.ac.uk/radon/hpa-bgs.html). Details on who will be granted access to the data, and whether there will be a charge for data access can be found online.

ALSPAC data access is through a system of managed open access. Full details of all available data can be accessed through a fully searchable data dictionary provided on the ALSPAC study website ( http://www.bris.ac.uk/alspac/researchers/data-access/data-dictionary) and the steps below highlight how to apply for access to both the data included in this data note and all other ALSPAC data. The datasets presented in this data note are linked to ALSPAC project number B645; please quote this project number during your application. The ALSPAC variable codes highlighted in the dataset descriptions can be used to specify required variables.

1. Please read the ALSPAC access policy (PDF, 627kB) which describes the process of accessing the data and samples in detail and outlines the costs associated with doing so.
2. You may also find it useful to browse our fully searchable research proposals database, which lists all research projects that have been approved since April 2011.
3. Please submit your research proposal for consideration by the ALSPAC Executive Committee using the online process. You will receive a response within 10 working days to advise you whether your proposal has been approved.

If you have any questions about accessing data, please email alspac-data@bristol.ac.uk.

The ALSPAC data management plan describes in detail the policy regarding data sharing, which is through a system of managed open access.

### Extended data

Open Science Framework: Radon ALSPAC. https://doi.org/10.17605/OSF.IO/KGCHQ ^38^.

This project contains the following supplementary tables:

• Radon exposure distributions for population subsets of each EWAS model.
• Binary covariate distributions of population subsets of each EWAS model.
• Continuous covariate distributions of population subsets of each EWAS model.

Extended data are available under the terms of the Creative Commons Zero “No rights reserved” data waiver (CC0 1.0 Public domain dedication).

### Reporting guidelines

STROBE Guidelines for cohort studies have been used for this publication. DOI: https://doi.org/10.17605/OSF.IO/KGCHQ ^38^.
