# Supplementary material for: Residential exposure to radon and DNA methylation across the lifecourse: an exploratory study in the ALSPAC birth cohort
Source: Wellcome Open Res. 2019 Apr 15;4:3. Originally published 2019 Jan 7. [Version 2] doi: 10.12688/wellcomeopenres.14991.2 (PMC6426102; doi:10.12688/wellcomeopenres.14991.2)
Supplement: Peer review history

# Peer review: Reviewer response for version 1

**DOI:** 10.21956/wellcomeopenres.16353.r34584

**Published:** 2019-03-22

**Authors:** Mark P. Little

**Affiliations:** 1 Radiation Epidemiology Branch, National Cancer Institute (NCI), National Institutes of Health (NIH), Bethesda, MD, USA

**General comments**

 This is a generally well written paper. However, the analysis is defective in a number of ways. In the first instance, arguably the wrong thing is being analysed. Probably cumulative radon exposure up to a given age is the variable more likely to be relevant than instantaneous radon exposure level; one would expect this to correlate with radon exposure rate fairly highly at young age, but much less well at older ages. This may have something to do with why the measures of the mothers in middle age found nothing. Analysis of radon using the 6-level probability-of-exposure-above-action-level metric throws away data and may reduce power. Analysis should use continuous radon concentration levels. Arguably the false discovery rate (FDR) threshold is set too high. Sensitivity analysis should be conducted using Bonferroni multiple-comparison correction of *p*-values rather than FDR.

 There should be more discussion of the plausibility of these findings, in particular the lack of internal consistency, associated with the fact that different CpG sites are found at different ages, and the lack of external consistency, associated with the fact that none of the sites identified in this MS are the same as sites previously identified after much higher-level radon exposures.

**Specific comments (page, column, line)**

 p.4 col. 2 l.7-10: It would be better to analyse radon using the mean radon concentration as a continuous variable, rather than as a categorical variable, recording percentages above the action level (200 Bq m ^-3^), which throws away data and will also reduce power. There is also a degree of arbitrariness in how one chooses the 6 groups. I assume that radon exposure is measured instantaneously at various ages. As above, arguably cumulative lifetime radon exposure up to the age at blood measurement may be the more relevant exposure measure. This should be evaluated, if possible.

 p.4 col. 2 l.11-14: It is not clear how this really helps assess measurement error. It should be regarded more as a type of sensitivity analysis.

 p.4 col. 2 l.-19: Is the 6-level radon variable described previously on this page really being analysed as a continuous variable? If so, this is extraordinarily unwise, as it will introduce non-linearities into the model. The radon concentration should either (as above) be analysed as a continuous measure (my preference), or if you are going to define this 6-level variable it must be analysed as a factor variable.

 p.4 col. 2 l.-18 - -16: To further collapse the radon exposures into a <5% vs >5% variable is arguably even more a degradation of this data. I don’t think this should be reported.

 p.4 col. 2 l.-12 - -5: It would be helpful for all these variables to indicate where this information came from. Presumably some (e.g., parental income, parental education, tobacco and alcohol consumption), are assessed via questionnaire, others (e.g., heaviness of traffic), possibly via linkages with other datasets. Also, the levels used to categorize these variables, where they are not simply linear variables in the model, needs to be defined.

 p.4 col. 2 l.-6: What is “equivalized income”?

 p.5 col. 1 l.16: An FDR of 0.20 is too high. A more conservative choice would be 0.05, or even 0.01, and at least the higher of these should be the default, with the 0.01 value (possibly also the 0.20 value) used for sensitivity analysis. Another sensitivity analysis that should be performed is to use Bonferroni multiple-comparison *p*-value adjustment, although this may be too conservative.

 p.5 col. 1 l.-16: As above, I don’t think this really assesses the impact of measurement error. It should be regarded more as a type of sensitivity analysis.

 p.5 col. 1 l.-6 - -3: The plausibility of there being a radon methylation effect at birth (implying transplacental transmission of radon) should be discussed. The fact that at different ages completely different CpG sites are affected also seems implausible, and again needs discussion.

 p.5 col. 2 l.17-21:  It should be pointed out that radon levels in this Chinese uranium miner study are likely to exceed those here by a substantial amount (by at least an order of magnitude, and probably more).

 p.5 col. 2 l.-11 - -5:  It is not the case that “Assuming measurement error in this case is non-differential, generally resulting in bias to the null … misclassification of radon exposure will affect all participants in a similar way and is unlikely to bias associations with DNA methylation”. First, these two sentences seem to imply different things (of bias and its lack)! If a classical error model is assumed then the associations would be biased towards the null (see R Carroll *et al* *Measurement error in non-linear models. A modern perspective* ^1^), as implied by the first (but not the second) of these sentences. If on the other hand a Berkson error model is assumed then estimates will be approximately unbiased, although the variance will be underestimated (Zhang *et al PLoS ONE* 2017 ^2^), as implied by the second (but not the first) sentence.

I have read this submission. I believe that I have an appropriate level of expertise to state that I do not consider it to be of an acceptable scientific standard, for reasons outlined above.

## References

[1] CirpianMRuppertDStefanskiLACarrollRJ: Measurement Error in Nonlinear Models: A Modern Perspective, Second Edition. *Chapman and Hall*.2006;

[2] ZhangZPrestonDLSokolnikovMNapierBADegtevaMMorozBVostrotinVShiskinaEBirchallAStramDO: Correction of confidence intervals in excess relative risk models using Monte Carlo dosimetry systems with shared errors. *PLoS One*.2017;12(4) : 10.1371/journal.pone.0174641 e0174641 10.1371/journal.pone.0174641 28369141PMC5378348

# Response

**Published:** 2019-04-11

**Authors:** Frank de Vocht

**Affiliations:** University of Bristol, UK

We thank the reviewer for his comments. We believe most comments have been addressed in response to the comments from the other reviewers. However, we would like to clarify two specific issues related to the data:

 1. Radon exposure was only ever available to us in the 6 classes, and therefore we were not able to conduct analyses using mean or cumulative radon concentrations as the exposure metric (despite agreeing in principle withe reviewer's comment).

 2. All other data used were available from the ALSPAC dataset, with no additional linkage to other datasets performed in this study.

# Peer review: Reviewer response for version 1

**DOI:** 10.21956/wellcomeopenres.16353.r34800

**Published:** 2019-03-19

**Authors:** Rayjean J. Hung

**Affiliations:** 1 Lunenfeld-Tanenbaum Research Institute, Sinai Health System, Toronto, ON, Canada

The authors investigated whether indoor radon exposure is associated with any DNA aberration based on maternal blood samples during pregnancy, coredblood, offspring’s peripheral blood samples age 7 and 15, within the ALSPAC birth cohort. They reported several different CpG sites to be associated with radon exposures at different time points based on FDR of 20% as threshold. The main comments are summarized below: 

1. ** Citing current literature: **The majority of the background section is radon exposure. While it is necessary to cover the property of radon exposures and measurements, more specific context related to methylation and radon, or more generally with ionizing radiation would be more informative for the readers. For example, animal models have showed ionizing radiations can result in transgenerational effects of DNA methylation (Kamstra et al, Sci Rep, 2018 ^1^; Trijau et al, Environ Sci Technol, 2018 ^2^, etc), and in vitro study showed that radon is related to aberrant DNA methylation in human lung cell lines. These papers can be provided as part of the background information. 
2. **Potential trans-generational effect:** With the multigenerational data in ALSPAC, one of the most interesting possibilities is to investigate whether radon exposures confer any trans-generational effects on methylation. Two of the relevant citations based on animal models were provided above. It would be valuable to assess if the methylation aberration induced by radon exposures persist across generations. 

**    **   3. **Statistical Analysis:  **

- The FDR of 20% as threshold seems rather generous. Most epigenomic analyses apply FDR of 10% or 5% as the threshold. When using 10% as the threshold, only cg25422346 from maternal blood during pregnancy remains to be significant.  
- The analysis is based on complete-case data. It would be helpful to know if the subset that was included in the analysis is representative of the whole population in terms of their main characteristics and radon exposures. Based on the tables, it seems that the main reduction of sample size occurs between Model 1 and Model 2. One could also consider conducting multiple imputations for the covariates used for adjustment in the model to minimize the loss of power.

**       ** 4. **Conclusions: **

- With the exception of cg25422346, the rest of the results have FDR larger than 10%. The authors did mention that this is an exploratory analysis. More caution on the relatively limited sample size can be emphasized.  
- While there are some interesting findings, none of the CpG sites overlap between any 2 time points, even when comparing the results of Age 7 and 15 of the offspring. One would expect that some methylation changes could persist over time. More discussions, either through the perspective of limited statistical power or biological interpretation, on the highly variable findings by time points would be warranted.
- Overall, it is not immediately clear what biological insights are gained from these results. Some more in-depth discussion on the implications of these results would be helpful. For examples, some regions were hypermethylated and some were hypomethylated. Whether these findings align with the biological functions of those genetic regions should be discussed. If there are no explicit mechanism related to these methylation cites, it would also be good to state so.

I have read this submission. I believe that I have an appropriate level of expertise to confirm that it is of an acceptable scientific standard.

## References

[1] KamstraJHHuremSMartinLMLindemanLCLeglerJOughtonDSalbuBBredeDALycheJLAleströmP: Ionizing radiation induces transgenerational effects of DNA methylation in zebrafish. *Sci Rep*.2018;8(1) : 10.1038/s41598-018-33817-w 15373 10.1038/s41598-018-33817-w 30337673PMC6193964

[2] TrijauMAsselmanJArmantOAdam-GuillerminCDe SchamphelaereKACAlonzoF: Transgenerational DNA Methylation Changes in Daphnia magna Exposed to Chronic γ Irradiation. *Environ Sci Technol*.2018;52(7) : 10.1021/acs.est.7b05695 4331-4339 10.1021/acs.est.7b05695 29486114

[3] SchofieldPNKondratowiczM: Evolving paradigms for the biological response to low dose ionizing radiation; the role of epigenetics. *Int J Radiat Biol*.2018;94(8) : 10.1080/09553002.2017.1388548 769-781 10.1080/09553002.2017.1388548 29157078

[4] HuangHJiYZhangJSuZLiuMTongJGeCChenTLiJ: Aberrant DNA methylation in radon and/or cigarette smoke-induced malignant transformation in BEAS-2B human lung cell line. *J Toxicol Environ Health A*.2017;80(23-24) : 10.1080/15287394.2017.1384156 1321-1330 10.1080/15287394.2017.1384156 29048996

# Response

**Published:** 2019-04-09

**Authors:** Frank de Vocht

**Affiliations:** University of Bristol, UK

We would like to thank the reviewer for the comments and suggestions, as well as for highlighting several additional literature references to us. We have addressed all comments as follows:

1. ** Citing current literature: **The majority of the background section is radon exposure. While it is necessary to cover the property of radon exposures and measurements, more specific context related to methylation and radon, or more generally with ionizing radiation would be more informative for the readers. For example, animal models have showed ionizing radiations can result in transgenerational effects of DNA methylation (Kamstra et al, Sci Rep, 2018 ^1^; Trijau et al, Environ Sci Technol, 2018 ^2^, etc), and in vitro study showed that radon is related to aberrant DNA methylation in human lung cell lines. These papers can be provided as part of the background information. 

RESPONSE: Thank you for pointing us to these references. We have included these, and some other additional ones, in our manuscript and have included a brief discussion of this evidence to the Introduction section:

*Although the mechanisms of radiation-induced changes in DNA methylation remain largely unknown , the most plausible mechanism that has been proposed describes the effects of radiation on DNA methyltransferases ^20^, while it has further been suggested that low dose radiation can increase DNA methylation at least in part through the generation of Reactive Oxygen Species (ROS) ^21,22^. Ionising radiation exposure has been shown to affect DNA methylation in in vivo studies, and have the potential to be transmitted via the germline to subsequent generations* *^23,24^. *

1. **Potential trans-generational effect:** With the multigenerational data in ALSPAC, one of the most interesting possibilities is to investigate whether radon exposures confer any trans-generational effects on methylation. Two of the relevant citations based on animal models were provided above. It would be valuable to assess if the methylation aberration induced by radon exposures persist across generations. 

RESPONSE: Given the limited sample size we do not think it is possible to study this in detail using this sample; especially since these exploratory analyses have not highlighted methylation effects on the same CpG sites or genes for mothers and children (similar to across different timepoints for children only). However, in response to comment 1 above, we have now added the possibility of transgenerational effects in the manuscript.

       3. **Statistical Analysis:  **

- The FDR of 20% as threshold seems rather generous. Most epigenomic analyses apply FDR of 10% or 5% as the threshold. When using 10% as the threshold, only cg25422346 from maternal blood during pregnancy remains to be significant.  

RESPONSE: We appreciate that an FDR of 20% was more generous than the traditionally used 10% (or 5%), but believe that because of the exploratory nature of this study it was more important to minimise false negative findings over false positive findings; the latter will need to be confirmed in future independent studies. We have added this to the Statistical methods section:

*Because of the exploratory nature of this study, associations at false discovery rate (FDR) less than 20% calculated using the q method* *^33^* *are reported instead of a more traditional 10% threshold.*

and Discussion section:

*And finally, because of the exploratory nature of this study we relaxed the FDR threshold for reporting of findings to 20% to minimise the probability of missing associations. The drawback of this choice was that some of our findings may have being false positives. Had a 10% FDR, more traditional in confirmatory studies, been used, only methylation at cg25422346 in mothers during pregnancy and of cg16260355 in adolescents would have been highlighted. *

- The analysis is based on complete-case data. It would be helpful to know if the subset that was included in the analysis is representative of the whole population in terms of their main characteristics and radon exposures. Based on the tables, it seems that the main reduction of sample size occurs between Model 1 and Model 2.

RESPONSE: We have added extended data tables exploring this, and have added the following to the manuscript:

*“* *We used complete-case analyses which resulted in different numbers of subjects included in the different models. However, assessment of the impact of case deletion indicated little differences between the different populations used for models 1-3, with the possible exception of smoking of the father for outcomes at birth an age 7 (Extended data:* *https://doi.org/10.17605/OSF.IO/KGCHQ* *).”*

- One could also consider conducting multiple imputations for the covariates used for adjustment in the model to minimize the loss of power.

RESPONSE: We addressed the issue of multiple imputations in the Discussion session: *“…* *we did not apply multiple imputation to increase sample size. In future, when feasible approaches have been developed, we plan to revisit these analyses.”*

        4. **Conclusions: **

- With the exception of cg25422346, the rest of the results have FDR larger than 10%. The authors did mention that this is an exploratory analysis. More caution on the relatively limited sample size can be emphasized.  

RESPONSE: We have now specifically added this as a limitation to our study in response to an earlier comment.

- While there are some interesting findings, none of the CpG sites overlap between any 2 time points, even when comparing the results of Age 7 and 15 of the offspring. One would expect that some methylation changes could persist over time. More discussions, either through the perspective of limited statistical power or biological interpretation, on the highly variable findings by time points would be warranted.

RESPONSE: We have now specifically added this point to the first paragraph of the Discussion section: *“* *However, none of these associations were observed at multiple time points.”*

However, there is an array of possible explanations for this, including the ones put forward by the reviewer, and because this is the first study looking at these associations at this stage it is not yet possible to favour one explanation over another. As such, we decided not to add a detailed discussion of this.

- Overall, it is not immediately clear what biological insights are gained from these results. Some more in-depth discussion on the implications of these results would be helpful. For examples, some regions were hypermethylated and some were hypomethylated. Whether these findings align with the biological functions of those genetic regions should be discussed. If there are no explicit mechanism related to these methylation cites, it would also be good to state so.

RESPONSE: This study adds to the knowledge of changes in methylation related to low dose radon exposure in normal, human, populations. Nonetheless, associations between ionizing radiation and epigenetic modifications is a new and active field of research in which the biological mechanisms have not yet been well described. There are several reviews discussing this (a recent one has now been included in this paper), and we now briefly highlight this in the Discussion:

*“Data from in vitro experiments has similarly shown both hypo-and hyper-methylation suggesting methylation status is probably dependent on the direct activity of the methylation machinery and could be mediated by the activity of associated DNA methyltransferases ^22^. Dose-dependent effects have also been demonstrated for global DNA methylation and LINE-1 in nuclear power plant workers ^39^.”*

# Peer review: Reviewer response for version 1

**DOI:** 10.21956/wellcomeopenres.16353.r34801

**Published:** 2019-02-20

**Authors:** Beata Peplonska

**Affiliations:** 1 Department of Environmental Epidemiology , Nofer Institute of Occupational Medicine, Lodz, Poland

The paper  entitled “ *Residential exposure to radon and DNA methylation across the lifecourse: an exploratory study in the ALSPAC birth cohort”* by Frank de Vocht et al. is a novel and stimulating contribution to studies on the effects of residential radon exposure. The study finds some significant associations between potential exposure to radon >200 Bq m3 and methylation pattern of cytosines within several CpGs  in children and one site in mothers. Both hypo- and hypermethylations have been detected.

 At the current stage of knowledge, the biological meaning of the detected epigenetic changes is not known, however, the work performed is still of value and the inferences observed may be clarified in the future research.

 The authors discuss their findings providing description of the pathologies related to the mutations of the genes  where the specific hypo- or hypermethylation has been found (lacking reference). Nevertheless, the epigenetic effects observed were neither located at the promoter sites of these genes nor enhancers, thus may not affect the genes activity, which in my opinion shall be stated explicitly. 

 The limits of the study, including lack of precise data on study participants exposure have been identified and described by the authors.

 The paper is very clearly written and reads very well.

 I have only few minor comments:

 Introduction:

 “Residential radon exposure has been associated with DNA repair gene polymorphisms in adults ^16^ and children ^17^, with the latter study also reporting double-strand break repair gene polymorphisms.”. Please, check in the cited articles what associations have been reported, and correct the statement accordingly.

 Methods:

 2 ^nd^ paragraph reports at which ages blood was collected from study subjects (children and mothers). The information for children is unnecessary repeated in the subsection describing “DNA methylation”.

I have read this submission. I believe that I have an appropriate level of expertise to confirm that it is of an acceptable scientific standard.

# Response

**Published:** 2019-04-09

**Authors:** Frank de Vocht

**Affiliations:** University of Bristol, UK

We would like to thank the reviewer for the comments and suggestions. 

We have addressed the two minor comments as follows:

“Residential radon exposure has been associated with DNA repair gene polymorphisms in adults ^16^ and children ^17^, with the latter study also reporting double-strand break repair gene polymorphisms.”. Please, check in the cited articles what associations have been reported, and correct the statement accordingly.

*RESPONSE: Residential radon exposure has been associated with DNA-repair gene polymorphisms in adults* *^16^* *and children* *^17^* *, with the latter study also reporting double-strand break repair gene polymorphisms.*

Has been changed to:

“ *Residential radon exposure has been associated with DNA-repair gene polymorphisms in adults (XpG gene Asp1104His, ADPRT gene Val762Ala, and NBS1 gene Glu185Gln polymorphisms) ^16, ^and partly replicated in children (XpD gene Lys751Gln, XpG gene Asp1104His and ADPRT gene Val762Ala polymorphisms)* *^17^* *, with the latter study also reporting double-strand break repair gene polymorphisms.”*

Methods: 2 ^nd^ paragraph reports at which ages blood was collected from study subjects (children and mothers). The information for children is unnecessary repeated in the subsection describing “DNA methylation”.

*RESPONSE: Thank you very much for highlighting this, we have removed this from the “DNA methylation” section.*
